# Assessment of the Antioxidant/Hypolipidemic Relationship of *Sideritis hyssopifolia* in an Experimental Animal Model

**DOI:** 10.3390/molecules24112049

**Published:** 2019-05-29

**Authors:** Esther Coto, Nelida Fernandez, Juan Jose Garcia, M. Jose Diez, Ana Maria Sahagun, Matilde Sierra

**Affiliations:** Pharmacology, Institute of Biomedicine (IBIOMED), University of Leon, 24071 Leon, Spain; esther.coto@gmail.com (E.C.); jjgarv@unileon.es (J.J.G.); mjdiel@unileon.es (M.J.D.); jmrodl@unileon.es (A.M.S.); msiev@unileon.es (M.S.)

**Keywords:** antioxidant activity, atherogenic index, cholesterol, rabbit, *Sideritis hyssopifolia*

## Abstract

Many publications have described the potential cardioprotective action of different medicinal plants, relating this effect with blood lipid levels. However, these publications do not justify the right amount of plant administered, which can vary greatly. *Sideritis hyssopifolia* is a little woody plant endemic to western and southwestern Europe. We have quantified its antioxidant activity, which can be used as an indicator of its cardioprotective action. This study evaluates the antioxidant capacity of *Sideritis hyssopifolia* to design a feed whose hypolipidemic effects are proven in cholesterol-fed New Zealand rabbits. Antioxidant action was assessed in infusions, which were prepared with 1 or 3 g of plant in 200 mL of water by using an ABTS assay and expressed as Ascorbic acid Equivalent Antioxidant Capacity (AEAC). Aqueous infusions with infusion times of 10 min and prepared with 3 g plant exhibited the strongest antioxidant activity. *Sideritis hyssopifolia* showed an intermediate antioxidant capacity for the concentrations and times of the infusion tested. According to our results, we suggest incorporating 2.36 g of *S. hyssopifolia* every 150 g of rabbit feeding stuff (15.73 g/kg). This chow decreased cholesterol, HDL-cholesterol, LDL-cholesterol, and triglycerides levels in cholesterol-fed rabbits, as well as the atherogenic index. This reduction was similar to that obtained with simvastatin.

## 1. Introduction

Atherosclerosis and its associated cardiovascular pathologies are the major causes of morbidity and mortality in Western countries, and represent one of the greatest worldwide economic, social and medical challenges that we are facing at the present time [1,2]. Hypercholesterolemia and its induced oxidative stress are considered one of the major contributors to the initiation and progression of atherosclerosis [3,4]. The initial arterial lesion, the fatty streaks formation and the development of atheroma plaques are closely linked with the accumulation of oxidized low-density lipoproteins (LDL) in macrophages. High lipid concentrations in plasma may alter lipoprotein metabolism, causing the accumulation of LDL in the subendothelial layer of the vessels, where these proteins undergo oxidative changes, resulting in oxidized LDL, a compound that is highly atherogenic [5,6,7,8]. Thus, oxidative modification of LDL is an important factor in atherosclerotic changes [6,7,8], whereas high density lipoproteins (HDL) play a key role in the protection against the oxidative damage of cells [9,10].

Even though there are several medications available to prevent the underlying causes of this disorder, adverse effects are consistently present. Thus, the development of additional therapies for controlling cholesterol levels is warranted, especially those with better safety profiles. In this way, in the last decades there has been a great interest in investigating the effectiveness and safety of lipid-lowering agents of natural origin obtained from medicinal plants.

Antioxidants obtained from these natural sources are increasingly being recognized as important health promoters in cardiovascular or neurodegenerative disorders [11,12,13,14]. Several authors have demonstrated that antioxidant intake is inversely related to the incidence of heart attacks and mortality from coronary heart disease [15,16,17].

Antioxidants suppress the development of hypercholesterolemic atherosclerosis, and induce its regression. This process is associated with decreases in oxidative stress and serum lipids [18,19]. Among antioxidants, polyphenols present in plants prevent LDL oxidation [20,21] and may significantly diminish the risk of mortality from cardiovascular diseases [22,23,24].

In recent years, the genus *Sideritis* L. (*Lamiaceae* family) has attracted the attention of the scientific community as it has shown a wide range of medicinal folk properties, such as being anti-infectious, anti-inflammatory, anti-rheumatic and anti-ulcer applications [25,26]. This genus includes at least 150 species of annual and perennial plants [27], widely distributed in the Mediterranean region, the Balkans or the Iberian Peninsula, but also in other regions. *Sideritis hyssopifolia*, L. is a little woody plant endemic to western and southwestern Europe, primarily in mountainous areas from northern Portugal through to western Switzerland and northern Italy. In Spain, this medicinal plant has been traditionally used since ancient times as tea due to its digestive properties and beneficial effects on the treatment of gastric ulcers. These properties have been attributed to the high polyphenolic content of these plants [28,29].

In this study, we evaluate the antioxidant activity of the medicinal plant *S. hyssopifolia*, and accordingly with this activity, we propose the preparation of a chow containing this medicinal plant at a certain amount, to carry out the experimental assessment of its cardioprotective action in a hypercholesterolemic rabbit model. In other studies, the authors have related the lipid-lowering action of different medicinal plants with their antioxidant activities, but they fail to justify the amounts used, which vary greatly in the different studies: 10 mg/kg [30] and 288 mg/kg [31] in the case of plant extracts, and 1000 mg/kg [32] and 13,000 mg/kg [33] for fresh plants or seeds.

Ascorbic acid is the most important vitamin present in fruits and vegetables, and it is involved in many physiological functions in human organisms, acting as the scavenger for oxidizing free radicals and harmful oxygen-derived species. As an antioxidant, it reduces the risk of suffering cardiovascular diseases, such as atherosclerosis, and some types of cancer [34]. In hypercholesterolemic patients, the administration of 2 g ascorbic acid decreased oxidative stress and improved endothelial dysfunction [35,36,37,38], probably by scavenging super-oxide anion and preventing inactivation of NO [36,39], as well as by the inhibition of LDL oxidation [40]. Therefore, a daily administration of 2 g ascorbic acid would have protective activity against cardiovascular diseases. 

The purposes of this study were to evaluate the antioxidant activity of *Sideritis hyssopifolia*, and to determine the amount of this plant that should be added to a rabbit feed in order to achieve a protective action against cardiovascular disorders in hypercholesterolemic rabbits, taking into account those data reported by several authors on the effectiveness of ascorbic acid [35,36,37,38]. The proposed chow could serve as guidance for other medicinal plants. The lipid lowering properties and the atheroprotective effect of *Sideritis hyssopifolia, L.* in high cholesterol fed New Zealand rabbits was then proved by using this chow.

## 2. Results

### 2.1. Antioxidant Properties

**Figure 1:**
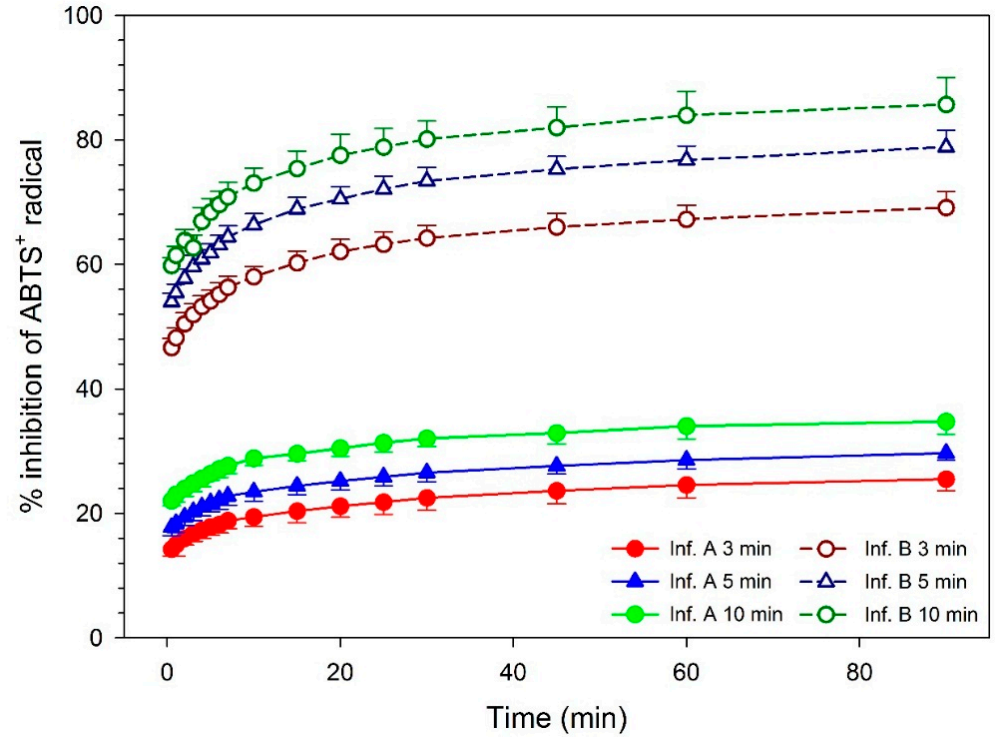
Antioxidant activity of *Sideritis hyssopifolia* infusions versus time. Antioxidant activity is expressed as inhibition percentage of ABTS^•+^ radical. Two different infusions (A: 5 × 10^−5^ g/mL; B: 1.5 × 10^−4^ g/mL), and three times of infusion were assayed (3, 5 and 10 min). Data are expressed as mean ± SD (n = 5).

Inhibition percentages versus time for infusions A and B after 3, 5, and 10 min of contact plant-water contact are provided in Figure 1. As shown in the figure, the longer the time of the infusion, the higher inhibition capacity and, consequently, the higher the antioxidant activity. Accordingly, in infusion A samples, an initial inhibition percentage of 14.24% was obtained with 3 min of infusion; 17.76% after an infusion time of 5 min; and 22.04% with 10 min. With infusion B samples, an initial inhibition percentage of 46.62% was calculated for an infusion time of 3 min, 53.97% with 5 min, and 59.81% with 10 min.

Moreover, antioxidant activity increased with the amount of plant added. Thus, in infusion B samples (1.5 × 10^−4^ g/mL), an inhibition percentage of 70.82% was obtained with an infusion time of 10 min, while it dropped to 27.64% with infusion A samples (5 × 10^−5^ g/mL) in the same conditions.

IC_50_ values were then calculated for ascorbic acid and infusions A and B (Table 1). Calculations were made with absorbance obtained at 7 min of reaction for ascorbic acid, and at 45 min for infusions A and B, as values remained constant after this reading. Ascorbic acid Equivalent Antioxidant Capacity (AEAC) values were also calculated for the different infusion times and amount of plant used, ranging from 1.808 to 2.419 g/100 g.

The antioxidant activity of 2 g ascorbic acid would be equivalent to 110.62 g of *S. hyssopifolia* after an infusion time of 3 min; 95.70 g of plant when an infusion time of 5 min was used, or 82.67 g with an infusion time of 10 min. Taking into account these results, if we add this plant to the feed of rabbits, the amount of antioxidant compounds available would be, at least, those found with the highest time of infusion (10 min). As the maximum extraction efficiency of antioxidant compounds was obtained with an infusion time of 10 min, we have used the value of 82.67 g (equivalent to 2 g ascorbic acid) to calculate the amount of *S. hyssopifolia* that should be added to the chow in order to achieve a protective action against cardiovascular disorders in hypercholesterolemic rabbits. A man (70 kg standard weight) would need 1.18 g/kg body weight [37]. Therefore, for a rabbit (2 kg mean weight), 2.36 g of plant should be included in its dairy feeding (150 g). Thus, we consider that 2.36 g of *S. hyssopifolia* should be added to every 150 g of feeding stuff (15.73 g/kg or 1.57% w/w) to assess its cholesterol-lowering effects in hypercholesterolemic rabbits.

### 2.2. Effects on Lipid Profile

No mortality and no clinical signs of toxicity were observed in the rabbits during the experimental period. All animals remained healthy and were suitable for the study.

Table 2 includes the mean values for the different parameters evaluated: total cholesterol, HDL-cholesterol, LDL-cholesterol, triglycerides, and atherogenic index at the beginning of the study and after 6 weeks of treatment with the different chows.

Cholesterol rich chow caused hyperlipidemia in all the animals. After 6 weeks of treatment, total cholesterol levels increased significantly in cholesterol, Sideritis and Simvastatin groups (*p* ≤ 0.05). However, this increase was approximately 1.2-fold lower in Sideritis and Simvastatin groups than in cholesterol group, although significant differences only were only found between cholesterol and Simvastatin groups (*p* ≤ 0.05).

HDL-c values were also higher at the end of the treatment in cholesterol, Sideritis and Simvastatin groups (*p* ≤ 0.05). Differences between these three groups were lower than for the total cholesterol. The levels determined for LDL-c also increased from week 0 to week 6 in the cholesterol, Sideritis, and Simvastatin groups (*p* ≤ 0.05). This parameter showed significant differences between the cholesterol group and Sideritis and Simvastatin groups (*p* ≤ 0.05). The increase observed in triglycerides after receiving the diet was lower than that seen in cholesterol, HDL-c and LDL-c. The rise in triglyceride levels was also lower in the Sideritis and Simvastatin groups than in the cholesterol group, with no significant differences between them.

The highest value for the atherogenic index was obtained in the cholesterol group at the end of the treatment (significant differences between cholesterol group and Sideritis and Simvastatin groups; *p* ≤ 0.05).

## 3. Discussion

We have determined the antioxidant activity of this plant from infusions, which is the major way of incorporating this medicinal herb to a diet. To our knowledge, this is the first time that the antioxidant activity of a medicinal plant has been correlated to the amount of the plant that may be added to a feed or diet to demonstrate its hypocholesterolemic activity. According to the literature reviewed, other authors have used different medicinal plants to study their lipid-lowering effects in animal models [30,32,41,42,43,44], supplementing an animal diet with a certain amount of the plant or extracts of the plant, but these studies do not clearly associate the amount added with the antioxidant activity of the plant.

The intake of food with an antioxidant effect has been associated with a low risk of cardiovascular diseases [45]. In our study, the antioxidant activity of *S. hyssopifolia* was assessed after three different infusion times (3, 5 and 10 min). Our results showed that the longest infusion time (10 min) exhibited stronger AEAC values (and antioxidant activity) than the other two infusion times (3 and 5 min).

*S. hyssopifolia* exhibited an intermediate antioxidant capacity when comparing the AEAC values obtained in our study with those indicated by other authors. Thus, the AEAC values were higher in fresh plants of the *Zingiberaceae* family such as *Alpinia zerumbet* (3020 mg/100 g) or *Etlingera elatior* (2990 mg/100 g) [46], in several tropical and temperate herbal teas of lemon myrtle (*Backhousia citriodora*) (13,600 mg/100 g), guava (*Psidium guajaba*) (7430 mg/100 g), rosemary (*Rosmarinus officinalis*) (3090 mg/100 g) or mint (*Mentha spicata*) (4430 mg/100 g) [47], or in medicinal plants of *Leguminosae* family, which exhibited AEAC values for flowers and leaves, respectively, of 14,600 and 6410 mg/100 g for *Bauhinia kockiana*, 3350 and 7690 mg/100 g for *Caesalpinia pulcherrima*, and 4080 and 4130 mg/100 g for *Cassia surattensis* [48]. Nevertheless, other plants or teas clearly showed AEAC values clearly lower than those calculated by us, such as *Curcuma longa* (251 mg/100 g) or *Kaempferia galangal* (48 mg/100 g) [46], as well as teas of chamomila (*Matricaria recutita*) (966 mg/100 g) [47].

Flavonoids, ditherpenes and essential oils are present in the *Sideritis* species, which endow these plants with important antioxidant, anti-inflammatory, anti-ulcer, antimicrobial and analgesic properties [27]. Nine 7-ortho-alosil glycosides have been identified by spectroscopic methods from the methanol extract of the aerial part of *Sideritis raeseri*, conferring these components gastroprotective, anti-inflammatory and antioxidant characteristics [49]. Regarding *S. hyssopifolia*, it is known to contain four flavone glycosides, which are supposed to have antioxidant properties [50].

The hypolipidemic effect of the plant, included in the chow at the concentration determined after the evaluation of its antioxidant effect, was determined in cholesterol-fed New Zealand rabbits. Due to the high impact of atherosclerosis on health, several animal models have been used as tools to investigate dyslipidemia and the pathophysiology of this disease. Nowadays, rabbits are considered the main pre-clinical model to carry out these studies because this animal model is more sensitive to experimental atherosclerosis, which can be viewed after one week of exposure [51].

The anti-hypercholesterolemic effects of *Sideritis hyssopifolia* has been demonstrated in this study for the first time. Other authors have demonstrated the antioxidant properties of different Sideritis species [52,53] as well as their lowering effect on triglycerides [54]. The antioxidant activity found for these species was intermediate, as our results indicate for *Sideritis hyssopifolia*, L. This antioxidant activity has been related mainly to their content in polyphenols. Soluble fibers obtained from different plants have been also related to cholesterol lowering effects, but the amounts that need to be administered daily are much higher than those provided by the addition of *Sideritis hyssopifolia* to the chow.

In our research, the addition of 2% of cholesterol in the diet of rabbits caused an increase in all the parameters evaluated. The overall results of our study reveal that *Sideritis hyssopifolia*, L. in the dose added to the chow (2.36 g *Sideritis hyssopifolia*, L./150 g chow), has very similar hypolipidemic effects to simvastatin in the dose of 1,2 mg/kg/day, which may be of value in cardiovascular diseases. The total cholesterol, HDL-c, LDL-c and triglyceride values were lower in the Sideritis and Simvastatin groups than in cholesterol group, although only LDL-c showed significant differences. We think that, although Sideritis and Simvastatin showed a protective effect, the high levels of cholesterol reached with the diet, didn´t allow one to maintain the levels of the different parameters similar to those determined in the control group, even though these levels were smaller than those of cholesterol group.

The atherogenic index also showed significant differences between the cholesterol group and the Sideritis and Simvastatin groups. Further studies are required to determine the long-term effects of *Sideritis hyssopifolia*, L. on atherosclerosis and to evaluate the components responsible of this action.

## 4. Materials and Methods

### 4.1. Evaluation of the Antioxidant Properties

#### 4.1.1. Reagents

ABTS (2,2’-azinobis (3-ethylbenzothiazoline-6-sulphonic acid) di-ammonium salt) (Sigma-Aldrich, St. Louis, MO, USA), potassium persulfate (Sigma-Aldrich, St. Louis, MO, USA), L-ascorbic acid (Sigma-Aldrich, St. Louis, MO, USA), methanol (Merck, Darmstadt, Germany), and distilled water were used. All reagents were of analytical grade.

#### 4.1.2. Plant Material and Preparation of Sideritis Infusions

The aerial part of the plant was collected from the Picos de Europa, mountain chain that runs along Northern Spain. The plant was identified by Dr. Matilde Sierra. A sample of the plant was deposited in the herbarium at the University of Leon (LEB-203581). *S. hyssopifolia* infusions were obtained from the aerial part of the plant, following the instructions described previously [55]. Plants were cut into pieces that were homogenized to obtain a mixture with the same proportion of flowers and stalks. Later, portions of 1 g or 3 g of this plant material were weighed and stored until their use. Two-hundred milliliters of boiling water were added to 1 g (infusion A: 0.005 g/mL) or 3 g (infusion B: 0.015 g/mL) of plant material. Infusions were allowed to steep for 10 min with continuous swirling. Samples were then obtained at 3, 5, and 10 min.

#### 4.1.3. Determination of Antioxidant Activity

Antioxidant activity of aqueous extracts was measured using the 2,2’-azinobis-(3-ethylbenzothiazoline-6-sulfonic acid) diammonium salt) radical cation (ABTS•+) decolorization assay [56].

Final concentrations measured in the assay were 5 × 10^−5^ g/mL in infusion A and 1.5 × 10^−4^ g/mL in infusion B. Absorbance measurements of the infusion samples (A sample) were made at 734 nm at 0.5, 1, 2, 3, 4, 5, 7, 10, 15, 20, 25, 30, 45 and 90 min. Samples were assayed in quintuplicate.

The inhibition percentage (p) of free radical formation was calculated as in equation:

(1) $$p\;=\;\frac{A_{\mathrm{ABTS}}{-A\;}_{\mathrm{sample}}}{A_{\mathrm{ABTS}}\;}\;\times \;100$$

Ascorbic acid was used to calculate a standard curve (0.5, 1, 1.5, 2, 2.5, 3, and 3.5 µg/mL). Absorbance measurements were made at 0.5, 1, 2, 3, 4, 5, 6, 7, 10, 15 and 20 min, and each sample was conducted in triplicate. Ascorbic acid inhibition percentages were plotted against concentration, and the calibration equation calculated was Y = −1.397 + 25.377X (R^2^ = 0.9936), where Y is the inhibition percentage and X is ascorbic acid concentration in µg/mL.

The radical scavenging activity of aqueous infusions was expressed as Ascorbic acid Equivalent Antioxidant Capacity (AEAC) in milligrams of ascorbic acid per 100 g of plant (mg/100 g) [42], which was calculated according to the Equation (2):

(2) $$\mathrm{AEAC}=\frac{{\mathrm{IC}}_{50}\;\mathrm{ascorbic}\;\mathrm{acid}}{{\mathrm{IC}}_{50}\;\mathrm{infusion}}{\;\times \;10}^{5}$$

where IC_50_ is the concentration of ascorbic acid or infusion (IC_50_ infusion) that inhibits free radical formation by 50%.

### 4.2. Evaluation of the Effects on Lipid Profile

#### 4.2.1. Animals

Twenty-four healthy New Zealand white rabbits weighing 1.75–2.35 kg from the Animal Housing of the University of Leon were used. The animals were housed in individual metal cages, which allowed the isolation of feces in a lower container to avoid coprophagia. The environmental conditions were: humidity (55 ± 10%), temperature (19 ± 2 °C) and 12 h light-12 h dark cycle. Rabbits were maintained under these conditions at least 1 week before the assay, with free access to water and standard laboratory chow.

The rabbits were randomly divided into four groups of 6 rabbits each. The first group, (*Control* group) received a standard laboratory chow, whose composition was: proteins 16.5%, fats 3.4%, fiber 15.5%, ashes 7.2%, carbohydrates 19.3%, sugars 4.5%, vitamin A 15,000 UI/kg, vitamin D3 1100 UI/kg, vitamin E 100 mg/kg, vitamin K 3.5 mg/kg and copper 5 mg/kg (Ssniff, Germany). The second one (*Cholesterol* group) was fed with the same standard laboratory chow but enriched with 0.2% of cholesterol.

The third group (*Sideritis* group) received the standard laboratory chow enriched with cholesterol 0.2% and the plant *Sideritis hyssopifolia*, L. (2.36 g *Sideritis hyssopifolia*, and L./150 g of standard chow, as calculated previously).

The fourth group (*Simvastatin* group) was fed with the standard laboratory chow enriched with 0.2% of cholesterol and 20 mg/kg of simvastatin (Ferrer Pharma, Spain).

Animals received 150 g of laboratory chow every day during 6 weeks. Before feeding the animals, it was assessed if they had eaten all the administered fraction of food. The study was approved by the Ethics Committee of the University of León (Spain) with the reference number ETICA-ULE-002-2016.

#### 4.2.2. Blood Sampling

Blood samples (8–9 mL) were obtained from the marginal ear vein at the beginning (week 0), and at week 6 of the study. Immediately after collection, plasma was separated by centrifugation and stored at −80 °C until analyzed.

Total cholesterol (TC), high density lipoprotein cholesterol (HDL-c), low density lipoprotein cholesterol (LDL-c) and triglycerides (TG) in the plasma samples were determined by using specific kits (Chemicals diagnostic kits). All parameters were quantified by a spectrophotometer UV/VIS (Hitachi). The atherogenic index (IA) was calculated with the mathematic formula described by Hassan and Abdel-Wahhab [57].

#### 4.2.3. Statistical Evaluation

All parameters measured were determined for each animal and presented as arithmetic mean ± standard deviation (mean ± SD). Data were analyzed using the Skewness test (to determine the normality) and Cochran test (to determine the uniformity of the variance).

When the data were normal and there was uniformity in the variance, analysis of variance (ANOVA) was carried out and the Duncan test was used to determine differences between data sets. When the data were not normal and/or there was not uniformity in the variance, the Friedman test was employed and the Wilcoxon test was used to determine differences between data sets. *P* ≤ 0.05 was used as the level of significance for all analyses.

## 5. Conclusions

We have demonstrated that *Sideritis hyssopifolia* exhibits a moderate antioxidant activity and could be used as an accessible source of natural antioxidants. We have also calculated the amount of plant that may exhibit a hypocholesterolemic effects in a rabbit experimental model, which will help to evaluate its potential activity against atherosclerosis. The study carried out using the experimental animal model showed that this chow decreased cholesterol, HDL-cholesterol, LDL-cholesterol and triglyceride levels in cholesterol-fed rabbits, as well as the atherogenic index. This reduction was similar to that obtained with simvastatin.

## Figures and Tables

**Table 1:** IC_50_ and Ascorbic acid Equivalent Antioxidant Capacity (AEAC) values of *Sideritis hyssopifolia*.

	IC_50_ (µg/mL)	AEAC (g/100 g)
Ascorbic acid	2.025 ± 0.095	-
*Sideritis hyssopifolia*		
3 min	112.1 ± 4.0	1.808 ± 0.068
5 min	96.9 ± 2.9	2.090 ± 0.063
10 min	83.7 ± 2.5	2.419 ± 0.071

**Table 2:** Lipid profile and atherogenic index (mean ± SD) of the different groups (n = 6) at the beginning and at the end of the treatments (6 weeks).

	0 Weeks				6 Weeks			
Groups	Control	Cholesterol	Sideritis	Simvastatin	Control	Cholesterol	Sideritis	Simvastatin
**TC (mg/dl)**	89 ± 10	84 ± 12	86.1 ± 9.4	83 ± 11	81.8 ± 8.8	460 ± 67	366 ± 61	348 ± 62
**HDL-c (mg/dl)**	24.3 ± 3.1	23.8 ± 2.9	25.2 ± 3.7	25.8 ± 3.4	22.6 ± 3.9	58.2 ± 7.4	49.4 ± 9.6	49.1 ± 5.1
**LDL-c (mg/dl)**	47.9 ± 9.1	50.2 ± 8.6	5 ± 13	48.8 ± 9.4	46.4 ± 8.7	179 ± 23	148 ± 20	133 ± 22
**TG (mg/dl)**	101 ± 15	105 ± 13	99 ± 19	103± 16	97 ± 17	173 ± 19	146 ± 13	137 ± 15
**AI**	2.43 ± 0.14	2.61 ± 0.16	2.37 ± 0.15	2.54 ± 0.12	2.58 ± 0.14	7.06 ± 0.53	4.01 ± 0.32	3.37 ± 0.21

TC: total cholesterol; HDL-c: high density lipoprotein cholesterol; LDL-c: low density lipoprotein cholesterol; TG: triglycerides; AI: atherogenic index; SD: standard deviation.
